# Clinical Profile of Ectopic Pregnancy in a Tertiary Care Center: A Five-Year Retrospective Study

**DOI:** 10.31729/jnma.v64i293.9296

**Published:** 2026-01-31

**Authors:** Deepa Chudal, Agya Shrestha, Rojina Manandhar, Santosh Thakur, Deepak Kharel, Usha Shrestha, Binita Basnet

**Affiliations:** 1 Department of Obstetrics and Gynaecology, Nepal Police Hospital, Maharajgunj, Kathmandu, Nepal; 2 Department of Pathology, Nepal Police Hospital, Maharajgunj, Kathmandu, Nepal

**Keywords:** *ectopic pregnancy*, *laparoscopic salpingectomy*, *maternal morbidity*, *Nepal*, *risk factors*

## Abstract

**Introduction:**

Ectopic pregnancy is a major cause of maternal morbidity and mortality in the first trimester. Delayed diagnosis often results in rupture, leading to life-threatening complications and adverse fertility outcomes. This study aimed to evaluate the incidence, risk factors, clinical presentation, and management of ectopic pregnancy in a tertiary care center in Nepal.

**Methods:**

A five-year retrospective observational study was conducted at Nepal Police Hospital, Kathmandu from March 1, 2020, to February 28, 2025. All confirmed cases of ectopic pregnancy were included. Data on socio-demographics, risk factors, clinical features, operative findings, and management were collected from hospital records. Descriptive statistics were applied.

**Results:**

Among 1,760 live births, 42 (2.38%) cases of ectopic pregnancy were identified. The mean age of the patients was 29.69±5.41 years, with the majority of cases occurring in women aged 20-35 years. Abdominal pain was the most common presenting symptom, reported in 30 (71.42%) patients. A history of abortion was the leading risk factor, noted in 14 (33.3%) cases. Tubal rupture was observed in 37 (88.09%) patients, and the ampullary region was the most frequent site, involved in 38 (90.47%) cases. All patients underwent surgical management, with laparoscopic unilateral salpingectomy being the most commonly performed procedure in 36 (85.71%) cases.

**Conclusions:**

The most common presenting symptom was abdominal pain in patients in the age group of 31-35 years. Most patient presented with ruptured ectopics.

## INTRODUCTION

Ectopic pregnancy defined as the implantation of a fertilized ovum outside the uterine cavity, remains a major cause of firsttrimester maternal morbidity and mortality, accounting for approximately 0.1% of maternal deaths in Asia.^12^ Its’ global incidence is rising with more than 95% of cases occurring within the fallopian tubes.^3^ Common predisposing factors include pelvic inflammatory disease, pelvic or tubal surgery, and assisted reproductive techniques such as in vitro fertilization.^4^

Despite the availability of advanced diagnostic modalities, including high-resolution transvaginal ultrasonography and quantitative serum β-hCG assays, diagnosis often remains challenging. The classical triad of amenorrhea, abdominal pain, and vaginal bleeding is frequently incomplete or atypical, making early clinical suspicion critical for timely management.^5^

Delayed presentation is common and may result in catastrophic complications. This study evaluates the incidence, clinical profile, and management of ectopic pregnancies to promote early diagnosis and reduce maternal morbidity and mortality.

## METHODS

This was a hospital-based retrospective, observational, descriptive study conducted in the Department of Obstetrics and Gynaecology at Nepal Police Hospital, Kathmandu, Nepal, a tertiary care referral center. The study included all patients diagnosed and managed for ectopic pregnancy during a five-year period from March 1, 2020, to February 28, 2025. Ethical approval for the study was obtained from the Institutional Review Committee of Nepal Police Hospital (Reference Number 38-02/2082) prior to data collection.

All cases with a confirmed diagnosis of ectopic pregnancy based on clinical evaluation, transvaginal ultrasonography, and/or intraoperative findings were included. Patients with uncertain diagnosis, incomplete medical records, or managed outside the hospital were excluded. Relevant data were retrieved from hospital records, operation theatre registers, and patient discharge summaries. Variables analyzed included demographic details (age, parity, and gravidity), risk factors (previous pelvic surgery, pelvic inflammatory disease, and assisted reproductive techniques), clinical presentation, diagnostic modalities, site of implantation, type of surgical or medical management, intraoperative findings, and outcome parameters.

Diagnosis was established using a combination of clinical features and diagnostic tests. Transvaginal ultrasonography was performed in all suspected cases to identify adnexal masses, absence of intrauterine gestational sac, or free fluid in the pelvis. Serum β-hCG levels were correlated with ultrasound findings when available. Patients were managed either surgically or medically based on hemodynamic stability, serum β-hCG levels, and sonographic criteria. Surgical management included laparoscopic or open salpingectomy, while selected stable patients received medical management with intramuscular methotrexate following standard protocols.

Data were entered into Microsoft Excel and analyzed using Statistical Package for the Social Sciences (SPSS) version 22. Descriptive statistics such as mean, standard deviation, frequency, and percentage were used to summarize quantitative and categorical variables.

Strict confidentiality was maintained throughout the study, and patient identifiers were removed before data analysis. The findings aim to highlight the clinical spectrum, diagnostic trends, and outcomes of ectopic pregnancy to support early recognition and timely intervention in similar tertiary care settings.

## RESULTS

During the study period, 42 (2.38%) cases of ectopic pregnancy were identified among 1,760 live births. The mean age of patients was 29.69±5.41 years, with most cases occurring in women aged 20-35 years 34 (80%). The cases in 31-35-year age group accounted for 14 (33.33%). In terms of gravidity, 21 (50%) were second gravidas, 10 (23.80%) in third gravidas and 6 (14.28%) in primigravida. A history of abortion was reported in 14 (33.33%) patients and previous abdominal surgery in 11 (26.19%) patients (Table 1). Clinically, abdominal pain was observed in 30 (71.42%), 6 (14.28%) presented with vaginal bleeding and 3 (7.14%) with both pain and bleeding (Table 2).

**Table 1:** Demographic characteristics, gravidity and risk factors of ectopic pregnancy (n=42).

Age (years)	n(%)
<20	2(4.76)
20-25	9(21.43)
26-30	11(26.19)
31-35	14(33.34)
36-40	6(14.28)
Gravidity	
Primigravida	6(14.29)
2^nd^ gravida	21(50.00)
3^rd^ gravida	10(23.81)
4^th^ gravida	5(11.90)
Risk Factors	
History of abortion	14(33.33)
Previous abdominal surgery	11(26.19)
Emergency contraception use	5(11.90)
Assisted reproduction	5(11.90)
Previous ectopic pregnancy	3(7.14)
Prior pelvic infection	2(4.76)
No risk factors	2(4.76)

During surgery, 37 (88.09%) cases of ruptured ectopic pregnancy was observed. The ampullary region of the fallopian tube was the implantation site in 38 (90.47%) of patients(Table 2).

Management was exclusively surgical. Unilateral salpingectomy was performed in 40 (95.23%) cases, and laparoscopy was the surgical approach in 36 (85.71%) patients (Table 2).

**Table 2:** Clinical presentation and operative findings of ectopic pregnancy (n=42).

Symptoms	n(%)
Amenorrhea	42(100.00)
Abdominal pain	30(71.43)
Vaginal bleeding	6(14.29)
Pain+bleeding	3(7.14)
Asymptomatic	2(4.76)
Shock	1(2.38)
Condition at Surgery	
Ruptured ectopic	37(88.09)
Tubal abortion	3(7.14)
Unruptured ectopic	1(2.38)
Organized ectopic	1(2.38)
Site of Implantation	
Ampullary	38(90.47)
Ovarian	1(2.38)
Isthmic	1(2.38)
Cornual	1(2.38)
Fimbrial	1(2.38)
Surgical Management	
Unilateral salpingectomy	40(95.23)
Partial oophorectomy	1(2.38)
Cornual resection	1(2.38)
Surgical Approach	
Laparoscopy	36(85.71)
Laparotomy	6(14.29)

## DISCUSSION

This five-year retrospective study demonstrates that ectopic pregnancy remains a significant contributor to maternal morbidity in Nepal, with an incidence of 2.38% and a strikingly high rupture rate of 88.1%. The majority of cases occurred in women aged 20-35 years, with abortion history as the leading risk factor, and the ampullary region as the most frequent site of implantation.

Surgical management was universal, with laparoscopic salpingectomy being the predominant approach. These findings highlight both encouraging advances, such as the widespread adoption of laparoscopy, and ongoing challenges, particularly the late presentation leading to rupture and the consequent reliance on salpingectomy with potential fertility implications.

The incidence observed in this study is comparable to reports from other South Asian centers, where incidence rates range from 1.5-3%. However, it is notably higher than those reported in high-income countries, where rates are often <1% due to better access to early antenatal care.^6-8^

The demographic profile revealed that the majority of cases occurred in women aged 20-35 years, with a mean age of 29.69 years. This concentration within the peak reproductive years aligns with findings by Gaskins et al.^9^ and can be linked to higher sexual activity during this period. Furthermore, advanced maternal age itself is a known risk factor, as it can adversely affect tubal function and oocyte transport.^10^

Multiparity was a predominant characteristic 36 (85.71%), a finding similar to other studies, ^11,12^ suggesting that a higher number of pregnancies may cumulatively increase exposure to risk factors such as pelvic infections or procedures. Among these risk factors, a history of previous abortion was the most common (33.33%) in our cohort, a result that echoes the findings of various studies from Nepal and India,^13,14^ and points to potential iatrogenic or post-procedural tubal damage.

The rupture rate in our cohort, 37 (88.1%) is alarmingly high compared to regional studies in India and Bangladesh, where rupture is reported in 60-75% of cases.^15^ In contrast, studies from developed nations demonstrate rupture rates below 30%, largely due to earlier diagnosis using transvaginal ultrasonography and serial β-hCG testing.^7,8^ This disparity underscores persistent diagnostic delays in our setting, reflecting both limited resources and delayed patient presentation.

The anatomical distribution of implantation sites in this study aligns closely with global data, where the ampullary region accounts for 70-90% of ectopic pregnancies.^3,9^ Rare cases of ovarian, cornual, isthmic, and fimbrial pregnancies were also observed, consistent with findings from larger cohorts worldwide. ^16, 17^

Our findings highlight the universal reliance on surgical management, with laparoscopy accounting for 85.7% of cases. This proportion is considerably higher than in many resource-limited settings, where laparotomy still predominates.^18^ The adoption of laparoscopy in our center represents an important institutional advancement, offering patients reduced operative time, shorter hospital stays, and faster recovery. Comparable adoption of minimally invasive approaches has been reported in higher-income countries, reinforcing the benefits of laparoscopic capacity-building in low-resource environments.^17, 18^

However, the overwhelmingly high rate of salpingectomy (95.2%) contrasts with international trends, where conservative options such as salpingostomy or medical management with methotrexate are increasingly employed in selected patients, especially those desiring future fertility. The reliance on salpingectomy in our study reflects the advanced disease stage at presentation, where tubal rupture and extensive damage necessitate definitive surgery.^19^

The predominance of salpingectomy raises significant concerns for future fertility. Loss of a fallopian tube reduces the probability of spontaneous conception and may increase reliance on assisted reproductive technologies. In high-income countries, more conservative interventions, including salpingostomy and methotrexate therapy, are considered in women with unruptured ectopic pregnancies to preserve reproductive potential.^20^ The absence of conservative management in our series underscores the urgent need for earlier diagnosis, which could allow for fertility-preserving options in selected cases.

This study highlights the key clinical and public health priorities in the care of women with ectopic pregnancy. Early and accurate diagnosis remains crucial in preventing serious complications and reducing maternal illness and death. Improving access to high-resolution transvaginal ultrasound and reliable serum β-hCG testing ,especially in peripheral and rural health facilities, can help detect ectopic pregnancies before rupture occurs. Clinicians should remain alert and consider ectopic pregnancy in any woman of reproductive age who presents with abdominal pain, missed periods, or abnormal vaginal bleeding, even when classic symptoms are not obvious.

Equally important is patient education. Encouraging women to seek medical attention early when they experience lower abdominal pain or a missed menstrual cycle can lead to timely diagnosis and safer management. Women at higher risk such as, those with a history of pelvic inflammatory disease, previous abortions, tubal or pelvic surgery, or assisted reproductive techniques should be offered early first-trimester screening. Whenever possible, preserving future fertility should be a priority, using medical treatment or conservative surgical approaches in stable, unruptured cases to ensure better reproductive outcomes.

The limitations of this study are its retrospective design and single-center setting, which may limit generalizability to broader populations. Incomplete documentation and missing data could have led to selection or reporting bias. Additionally, the absence of socioeconomic and fertility outcome data restricts the comprehensive assessment of long-term implications.

Future multicentric, prospective studies incorporating detailed socioeconomic and fertility outcome analyses are warranted. Establishing standardized diagnostic and management protocols and promoting early referral networks could further improve reproductive health outcomes and reduce preventable maternal deaths related to ectopic pregnancy.

## CONCLUSION

One-third of the patients were in the age group of 31-35 years while half of the patients were in the second gravida. One-third of the patients had history of abortion and abdominal pain was the most common clinical presentation. Most of the patient presented with ruptured ectopic pregnancy and ampulla was the most common site.
